# “Reevaluating social networking dependency: Academic achievement beyond quantitative indicators”

**DOI:** 10.1016/j.puhip.2026.100735

**Published:** 2026-01-13

**Authors:** Kadek Suhardita, I Wayan Widana, Rikas Saputra, Erfan Ramadhani, Putu Ari Dharmayanti, Sri Datuti, Rizky Ananda Pohan, Ulfah, I Dewa Ayu Eka Purba Dharma, Laily Tiarani Soejanto, Arizona

**Affiliations:** a Universitas PGRI Mahadewa, Denpasar, Indonesia; b Universitas Islam Negeri Raden Fatah Palembang, South Sumatra, Indonesia; c Universitas PGRI Palembang, Palembang, Indonesia; d Universitas Pendidikan Ganesha, Indonesia; e Universitas Mahasaraswati Denpasar, Indonesia; f Institut Agama Islam Negeri Langsa, Langsa, Aceh, Indonesia; g Universitas Islam Nusantara, Bandung, Indonesia; h Universitas PGRI Kanjuruhan, Malang, Indonesia

**Keywords:** Social Networking Dependency, Academic Achievement, Digital Literacy, Higher Education, Global Perspective, Multidimensional Indicators

Dear Editor,

The article titled [1] "The Impact of Social Networking Addiction on the Academic Achievement of University Students Globally: A Meta-analysis" presents a timely and significant addition to the conversation surrounding digital behaviors in higher education. The meta-analytic methodology provides essential clarity by integrating empirical evidence from various contexts, thereby affirming its importance in today's interconnected society. The article's strength is found in its capacity to offer a global viewpoint on how dependency on social networking may adversely affect academic performance. However, we contend that the existing framework necessitates reassessment, especially regarding its dependence on quantitative metrics as the exclusive indicators of academic success [2]. This correspondence aims to recognize the merits of the authors' research while promoting a more comprehensive dialogue that encompasses multidimensional aspects of academic achievement beyond conventional numerical indicators such as GPA or standardized test scores.

Our fundamental assertion is that the effects of social networking dependency cannot be comprehensively grasped through quantitative results alone. Contemporary academic achievement encompasses more than just grades or quantifiable performance; it also incorporates essential skills such as critical thinking, digital literacy, adaptability, and emotional resilience [3]. These aspects are crucial in a time characterized by swift technological advancements and global interconnectedness. While the article sheds light on the dangers of excessive use, it is equally crucial to highlight the contextual adaptability of students who may derive significant benefits from digital networks [4]. We contend that any effort to generalize findings across different cultures and educational frameworks must be undertaken with caution, considering the diverse pedagogical traditions and socio-cultural contexts. Thus, reassessing social networking dependency necessitates not only statistical precision but also an interpretive sensitivity to the varied realities of academic life across the globe.

The research appropriately emphasizes the harmful relationship between excessive use of social networking and academic success; nevertheless, significant gaps persist. For example, although the quantitative link is clear, the fundamental mechanisms such as motivational displacement, attention fragmentation, or cognitive overload remain inadequately examined [5]. Additionally, the structure of social media platforms, including algorithmic nudges, notification systems, and gamification elements, significantly influences the development of addictive behaviors. These technological features enhance both risks and opportunities: they can distract learners but also foster collaborative learning environments when managed effectively. Future investigations should delve into these dual effects more thoroughly, incorporating insights from behavioral science, platform design research, and educational psychology [6]. Such interdisciplinary inquiry will aid in determining whether dependency is intrinsically detrimental or contextually manageable, providing a more nuanced understanding of social networking's impact on student performance.

To further this discussion, we suggest a strategic plan that places social networking dependency within a context of responsible digital engagement. This necessitates an exploration of three key areas: personal regulation, institutional policy, and technological design [7]. On an individual basis, promoting digital self-regulation through psychoeducational initiatives can enhance students’ adaptability. At the institutional level, universities ought to incorporate digital literacy programs that emphasize both the advantages and disadvantages of social networking [8]. On the technological front, it is crucial to collaborate with platform developers to establish healthier online environments. Achieving these solutions requires a strong commitment from all stakeholders educators, policymakers, parents, and students must work together to develop sustainable practices. In the absence of such collective backing, attempts to reduce dependency will likely remain disjointed. Therefore, a comprehensive approach not only facilitates problem identification but also provides constructive avenues to harmonize academic success with digital engagement.

This commentary has consequences for both practice and research. Our viewpoint urges practitioners to embrace multifaceted approaches to student development and move beyond crude cause-and-effect presumptions [9]. We draw attention to the need for researchers to improve assessment instruments in order to capture comprehensive measures of success. Our opinion is unusual because it shifts the focus from "addiction and decline" to "dependency and adaptation," broadening the discussion beyond grades. Its critical integration of cultural, psychological, and technological factors is one of its strong points; on the other hand, its lack of direct empirical support from our end is one of its weaknesses. However, by placing social networking dependency in a more comprehensive, international, and multifaceted framework of academic success, the letter presents a forward-looking agenda that enhances the original article [10].

## Funding

The authors declare that no funding was received for this paper.

## Declaration of competing interest

The authors declare that they have no known competing financial interests or personal relationships that could have influenced the work reported in this paper.
